# Supplementary material for: Biological Control of a Root-Knot Nematode Meloidogyne incognita Infection of Tomato (Solanum lycopersicum L.) by the Oomycete Biocontrol Agent Pythium oligandrum
Source: J Fungi (Basel). 2024 Apr 2;10(4):265. doi: 10.3390/jof10040265 (PMC11051105; doi:10.3390/jof10040265)
Supplement: Supplementary file 1 [file jof-10-00265-s001.zip › Figure S1.pdf]

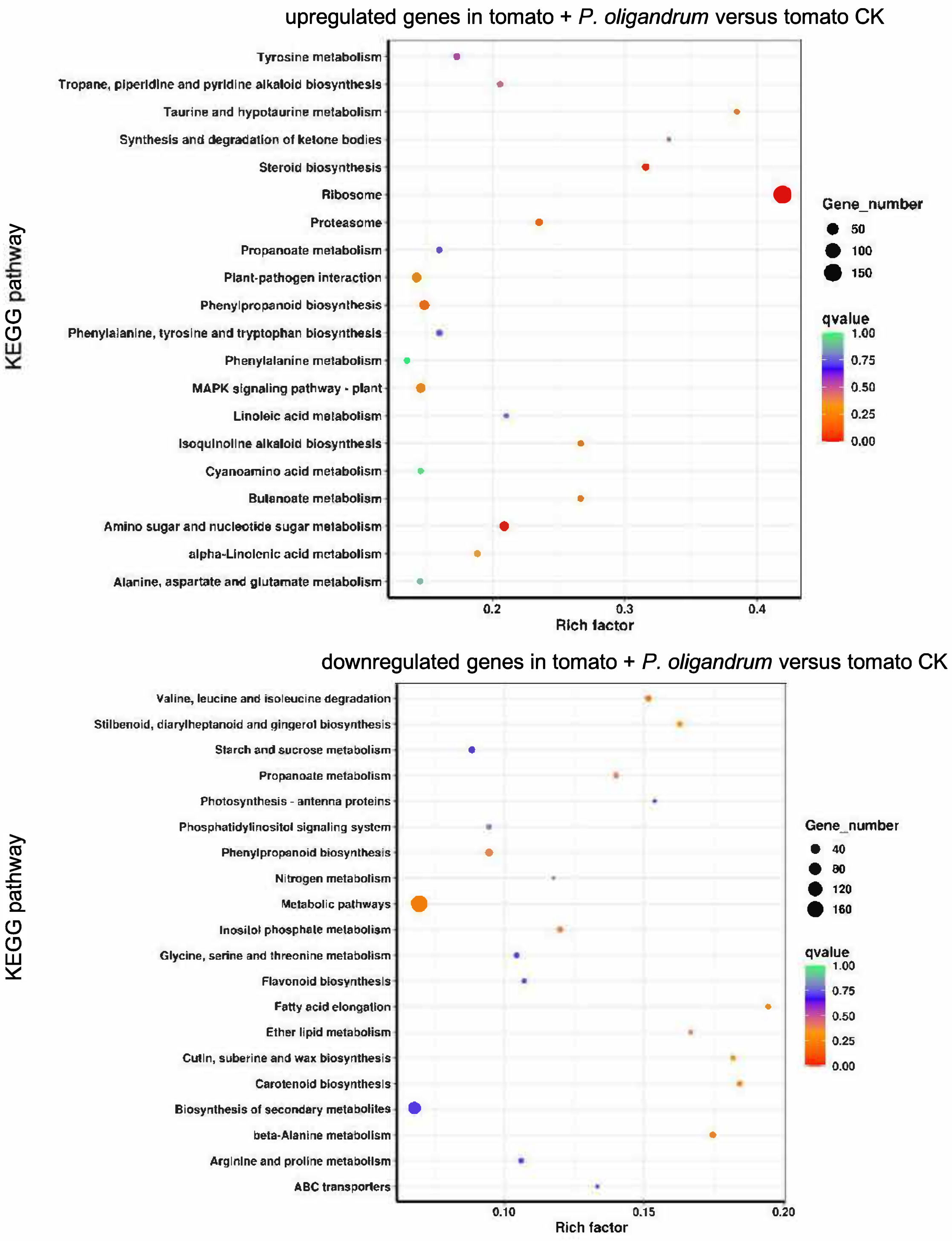


**Figure S1.** Overview of KEGG pathway enrichment from the RNAseq data of tomato seedlings roots with and without *P. oligandrum* GAQ1.
