# Supplementary material for: Biological Control of a Root-Knot Nematode Meloidogyne incognita Infection of Tomato (Solanum lycopersicum L.) by the Oomycete Biocontrol Agent Pythium oligandrum
Source: J Fungi (Basel). 2024 Apr 2;10(4):265. doi: 10.3390/jof10040265 (PMC11051105; doi:10.3390/jof10040265)
Supplement: Supplementary file 1 [file jof-10-00265-s001.zip › Figure S2.pdf]

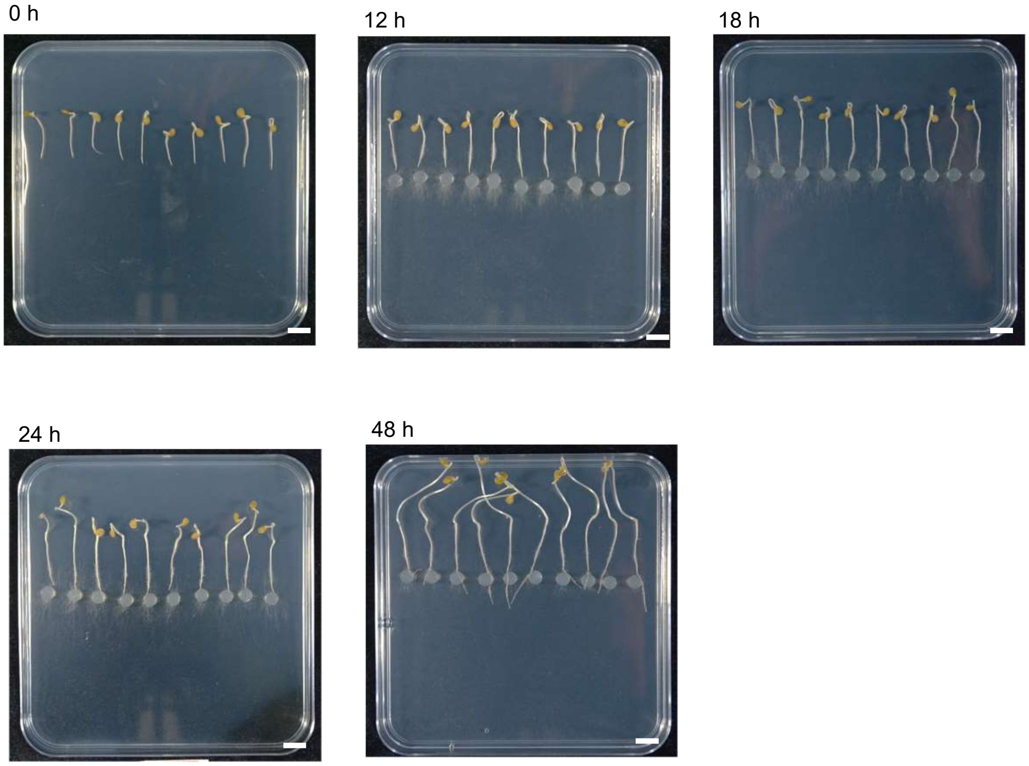

Figure S2. Representative images of tomato seedlings sampled for the time-course expression analysis before inoculation and at various time points after inoculation with agar plugs of *P. oligandrum* GAQ1. The scale bar corresponds to 1 cm.
