# Supplementary material for: Biological Control of a Root-Knot Nematode Meloidogyne incognita Infection of Tomato (Solanum lycopersicum L.) by the Oomycete Biocontrol Agent Pythium oligandrum
Source: J Fungi (Basel). 2024 Apr 2;10(4):265. doi: 10.3390/jof10040265 (PMC11051105; doi:10.3390/jof10040265)
Supplement: Supplementary file 1 [file jof-10-00265-s001.zip › Figure S3.pdf]

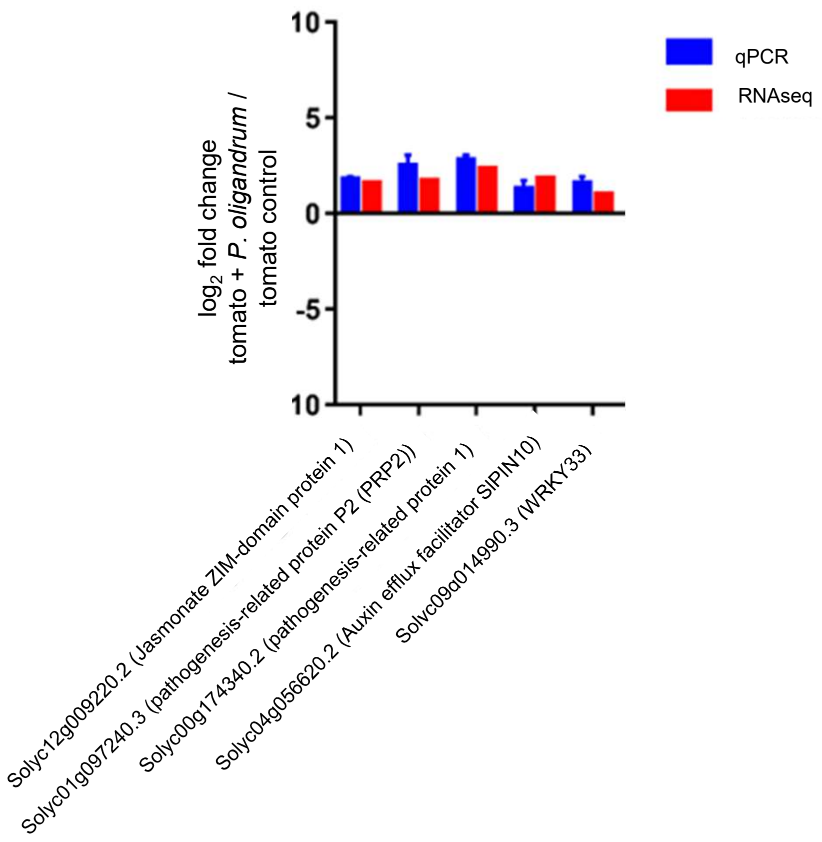

Figure S3. Fold changes of selected genes in tomato roots growing with *P. oligandrum* GAQ1 compared to control of tomato roots growing without *P. oligandrum* GAQ1 at 24 h. For the qPCR analysis, actin was used as the internal reference gene. The fold changes from qPCR are shown alongside the fold changes from RNAseq.
